# CFD Numerical Modelling of a PV–TEG Hybrid System Cooled by Air Heat Sink Coupled with a Single-Phase Inverter

**DOI:** 10.3390/ma14195800

**Published:** 2021-10-04

**Authors:** Artur Wodołażski, Natalia Howaniec, Bartłomiej Jura, Andrzej Bąk, Adam Smoliński

**Affiliations:** 1 Department of Energy Saving and Air Protection, Central Mining Institute, Pl. Gwarków 1, 40-166 Katowice, Poland; awodolazski@gig.eu (A.W.); nhowaniec@gig.eu (N.H.); 2 Department of Mining Aerology, Central Mining Institute, Pl. Gwarków 1, 40-166 Katowice, Poland; bjura@gig.eu; 3 Department of Organic Chemistry, Institute of Chemistry, University of Silesia, Szkolna 9, 40-007 Katowice, Poland; andrzej.bak@us.edu.pl; 4 Central Mining Institute, Pl. Gwarków 1, 40-166 Katowice, Poland

**Keywords:** photovoltaic module, thermoelectric generator, PV inverters hybrid system co-simulation

## Abstract

This study presents full transient, three-dimensional numerical models of a PV–TEG hybrid module coupled with single-phase inverter by co-simulation. The influence of factors, such as wind speed, solar radiation intensity, or ambient temperature on the PV–TEG system, was also examined. The numerical model was implemented using Ansys software which accounted the phenomena of Thomson, Seebeck, and Joule’s heat place on the TEG system. Furthermore, its impact on total electrical efficiency was studied. The heat transfer surface of the passive heat sink and forced air circulation positively affected the total heat transfer, and therefore helped to maintain the electrical efficiency at a higher level. Simulation of the single-phase inverter with a PV–TEG system allows the determination of the power characteristics of the system in real time. The results of the study presented may provide a basis for performance optimization of a practical PV–TEG-inverter hybrid system co-design.

## 1. Introduction

The vision of the climate neutrality by 2050 presented by the European Commission covers almost all European Union Policy areas and is consistent with the purpose of the Paris Agreement, which is to keep the temperature rise well below 2 °C and reduce it to 1.5 °C [1]. These strategies create a vision and set the direction of energy transformations. In order to mitigate the global climate change, an application of novel and economically sound, renewable-based energy resource technologies is crucial [2,3,4]. Solar energy is the most abundant of the renewable energy sources, which all reduce the dependency of energy systems on combustion of fossil fuels, causing photochemical or acidic smog and respiratory diseases [5,6,7]. The installed capacity of PV systems has been increasing steadily. PV systems may be used as an energy source in industrial plants, transport, and municipal heating systems. It reduces the carbon footprint of these systems contributing to the mitigation of the greenhouse gases emission [8,9,10,11].

Photovoltaics is the process of converting sunlight into electricity using solar cells. To characterize the incident solar energy, the following issues should be taken into account: the radiant power density from the sun, the spectral content of the incident light, as well as the angle at which the incident solar radiation strikes a photovoltaic module. Coupling of a thermoelectric generator (TEG) with solar panels of photovoltaic systems (PV) into PV–TEG improves the efficiency of such a hybrid system in terms of heat absorption. The p-n semiconductor devices are applied in conversion of the solar radiation energy into electricity [12]. The p-n semiconductor is bombarded with solar radiation photos of higher energy than the width of the semiconductor energy gap. This causes electrons and electron holes to move between the n and p regions of the semiconductor. The cyclical displacement of electric charges causes an increase in the potential difference, i.e., the electric voltage. This effect involves the emission of electrons from the surface of the object, while the kinetic energy of the photoelectrons emitted in this way does not depend on the intensity of the incident light, but on its frequency. Thus, there are photo electrochemical cells, which dissociates water molecules under the influence of sunlight. There are also methods that use photons for disinfection and detoxification. An area illuminated by sunlight in the direction perpendicular to its surface in the absence of cloud cover may receive approximately 800–1200 W/m^2^. The total energy that reaches the horizontal surface throughout the year is from 600 kWh/(m^2^/year) in the northern regions to over 2500 kWh/(m^2^/year) in the equatorial zone. In Poland, it is about 1100 kWh/(m^2^/year) [13]. For this reason, the efficiency of electrical conversion is only 14–22% of the heat dissipated, which causes an increase in the temperature of PV cells, reducing their efficiency. One of the crucial limit of PV systems is the low conversion efficiency of the PV panels which is significantly influenced by their operating temperature. Amelia et al. (2016) present that the conversion efficiency of the PV panels decrease by 0.40–0.50% for each degree rise in temperature [14]. PV electrical conversion efficiency is about 0.5%/°C for each degree rise in the PV panel temperature. Monocrystalline panels are characterized by efficiency of 15–24% and the temperature power factor of 0.43–0.50%/°C [2]. The efficiency of the polycrystalline panels is 14–18% and their temperature power factor is 0.40–0.47%/°C [15]. The electromagnetic radiation spectrum that supplies radiant energy to the PV systems consists of ultraviolet, visible light and infrared wavelengths where ultraviolet occupies 3% at 100–400 nm, visible light 44% at 400–700 nm and infrared 53% at above 700 nm. The relatively low conversion efficiency of solar energy into electricity means that further research efforts are needed to increase and stabilize their efficiency to make them less dependent on weather conditions and the variations in the intensity of solar radiation, impacting the current generated by the panel, and consequently its power. Fluctuations in radiation intensity do not significantly affect the voltage change in the photovoltaic panel thanks to the use of the maximum power point tracking (MPPT) which allows increasing the efficiency of the use of electricity generated by a PV module [16]. The active and passive cooling are the methods of increasing the efficiency of photovoltaic cells. The passive cooling technologies involve removing or minimizing the excess heat from the PV panel without consuming additional energy [17], with the use of metal fins of high thermal conductivity or other surface configurations to enhance dissipating overheating to the surrounding environment or phase change materials (PCMs) to transport heat to the environment employing a boiling-condensing process [18].

Another idea of converting thermal energy of the PVs systems into electricity is the use of a thermoelectric generators to make them widely applied, with the present state of efforts to improve their performance considering various factors, including temperature difference between hot- and cold-ends, geometry configurations (cross-section area, length, footprint), convection, load resistance, contact resistance, and fluctuations in the heat source [19]. Thermoelectric generators operate on the basis of the Seebeck phenomenon described in 1821 [20]. This effect involves the creation of a temperature difference on the side of a connection of two non-similar metals, with the voltage proportional to the temperature difference. The transformations of temperature differences based on Peltier and Seebeck effects are both employed in thermocouples. The dynamic properties of the system, however, affect the relationship between the amount of heat and electricity. The efficiency of the heat transport is the way to improve the overall electrical and thermal efficiency of the entire PV–TEG system [21]. The integration of PVs with TEGs enables an increase in power-generated and overall efficiency, when thermoelectric material with figure of merit of 0.004 K^−1^ leads to efficient enhancement of up to 23% [21,22] when compared to that of standalone PVs. Both density and heat capacity of air make the active cooling a promising technique, where mechanical devices such as air blowers or water pumping systems are installed to remove the overheating from front or back surfaces of the PV panels [22]. A hybrid PV–TEG system was first proposed by Van Sark [23], as a concept in which the waste heat energy of a photovoltaic cell may be utilized after connecting the TEG to the back of the PV module, resulting in the electrical efficiency increase to 23% for thermoelectric materials in the most favorable conditions. Chávez-Urbiola et al. [24] investigated a solar hybrid system with Bi3Ti4-based TEG for four different configurations with a temperature difference of 50–200 °C, showing that TEG performance, current, and voltage have a linear relationship with the temperature difference between the warm and cold ends of a TEG cell. Zhang and Chau [25] proposed and implemented a hybrid PV–TEG system for cars in which TEG was employed to utilize the waste heat of exhaust gas from a gasoline engine and optimize output power at maximum power point tracking. The results showed that the electrical and thermal efficiency of the PV–TEG panel increased from 16.7 to 23.5%. Yang and Yin [26] analyzed a PV–TEG hybrid system using water pipelines as a heat sink. The conversion efficiency depends on water temperature, solar radiation, and ambient temperature for the given material properties of each layer. The PV-TEC-TEG system is also considered in the literature [27]. Chen et al. [28] used the finite volume method to investigate the performance of a miniature thermoelectric cooler without taking the Thomson’s effect into account. The specific thermal profiles of the TEG module were obtained as a step response of a power semiconductor that can be measured experimentally or numerically simulated [18]. One of the great advantages of the numerical method is the ability to estimate the transient temperatures of all internal layers and the connections between them [29]. The efficiency of the energy transformation depends not only on the PV–TEG system but also on the electric circuitry (such as a three-phase inverter) connected to the PV–TEG module. To achieve the maximum power output, one needs to take into account the mutual influence of both components: PV–TEG and electric circuitry to design a system-level simulation, so-called a co-simulation. A co-simulation is a promising strategy to couple different parts of sub-systems concurrently. It is applied in multi-domain and multi-scale simulations, simultaneously coupling different types of sub-domains (mechanical, hydraulic, thermal and electrical), allowing for rapid prototyping and layout design for many specific cases of the coupled-domain behavior. A co-simulation couples thermoelectric computational fluid dynamics (CFD) and a circuit electric Simplorer domain, which demands many computational time and software resources. It is difficult to take into account TEG’s related Seebeck, Joul, or Thompson effects since the relevant electricity generated affects the voltage of the single-phase inverter and the way entire system affects each device. Therefore, these aspects were neglected in the literature on modelling electric domains with PV–TEG hybrid systems [30].

In the paper, a full transient CFD thermo-electric (PV–TEG) hybrid model, developed with the use of a co-simulation with single-phase PV inverters, to study computationally various approaches of integration synergy to improve electrical and thermal efficiency of PV–TEG systems cooled with air is presented. The proposed method of optimization of the inverters’ parameters, including the use of a “coupling approach”, enables easy design of such types of systems to optimize their performance, without having to lay down and solve partial differential equations (PDE). The proposed simulation model of an inverter is intended to be extended in the near future with additional parasitic elements of the inverter system. Furthermore, low-frequency transformers inverters (LF inverters) and high-frequency transformers inverters (HF inverters) will be also considered with PV–TEG to optimize system operation of a coupling method for an active cooling system with the advanced types of regulators, such as the balance-based adaptive control (B-BAC), internal model control (IMC), or dynamic matrix control (DMC) regulators.

## 2. Materials and Methods

According to the multi-physical field of the coupling Ansys electrothermal model of TEG and the CFD Fluent model of a solar panel (PV), a multi-level system co-design was created. The coupled PV–TEG model was employed in co-simulation with a single-phase inverter, which makes it difficult to analyze the problem with conventional methods. In order to carry out the multi-physical field coupling simulation, data information on the interactive Simplorer platform was established on the basis of the Ansys and Ansoft software (Ansys Inc., Canonsburg, PA, USA). Within the work presented, the electrothermal simulation elements of Ansys software version 15.0 were employed enabling for the simulation of thermal and electrical effects locally, with results covering the Joule, Seebeck, and Thomson effects.

### 2.1. PV Model

The temperature distribution in each layer of the photovoltaic module is governed by the following three dimensional energy equation:

(1) $$\rho \;c_{p}\frac{\partial T}{\partial t}-\nabla \cdot \left(\lambda \nabla T\right)=\dot{q}-P_{gen}$$

where: $c_{p}$—the specific heat capacity, ρ—the density, *T*—the temperature (K), $\dot{q}$—the volumetric solar energy absorption, $P_{gen}$—the electric power generation by volume, and λ—the thermal conductivity. The PV cell efficiency is calculated as follows [30]:

(2) $$\eta =\eta_{ref}(1-\beta \left(T_{PV}-T_{R}\right))$$

where $\eta_{ref}$—the reference cells efficiency; $T_{PV}$—the temperature of PV cells; $T_{R}$—the reference operating temperature; and β—the coefficient representing amount of efficiency loss per each temperature degree rise in the PV cells, i.e., (0.004 K^−1^). The volumetric solar energy absorption is calculated as follows:

(3) $$\dot{q}=\frac{\left[G_{i}\times \;\left(1\;-\;\alpha_{i}\right)\;-\;r_{i}\right]\;\times \;\alpha_{i}\times A_{i}}{V_{i}}$$

where $\alpha_{i}$, $r_{i}$, $V_{i}$ is the absorptivity, reflectivity, and volume of the layer, respectively; $G_{i}$ is the intensity received for each layer; and $A_{i}$—the exposed area of the PV cells.

The power generation of PV panel is:

(4) $$P_{gen}=\dot{q}\times \eta$$

Transient energy balance equation for the glass cover is:

(5) $$G\lambda_{gc}+Q_{PV\_GC}=d_{gc}\rho_{gc}C_{p,gc}\frac{\partial T_{gc}}{\partial t}+Q_{gca}+Q_{gcs}$$

where *G*—the solar radiation intensity; *λ_qc_*—the absorption rate of glass cover; *Q_PV-GC_*—the heat transfer between glass cover and photovoltaic cells; *Q_gca_*, *Q_gcs_*—the heat transfer between glass cover and PV cells, ambient air, and the surrounding environment.

The temperature of the glass cover is constant and each part of the cover receives the same amount of solar energy [31].

### 2.2. TEG Model

The equation of heat flow $\dot{q}$ in the TEG module is:

(6) $$\rho C\frac{\partial T}{\partial t}+\nabla \cdot q=\dot{q}$$

where C—the heat capacity, ρ—the density, $\dot{q}$—the heat generation rate per unit volume, ∇—the Nabla operator, and q—the heat flux density vector.

The equation of electric charge is:

(7) $$\nabla \cdot \left(J+\frac{\partial D}{\partial t}\right)=0$$

where *J*—the electric current density and *D*—the electric flux density vector.

Conjugated constitutive equations binding the phenomenon of thermoelectricity between the heat flux and the density of the electric current are expressed by the Formulas (8) and (9) [32]:

(8) $$q=\left[\Pi \right]\cdot J-\left[k\right]\cdot \nabla T$$

(9) $$J=\left[\sigma \right]\cdot \left(E-\left[\alpha \right]\cdot \nabla T\right)$$

where [Π]—the matrix of Peltier coefficient, $\left[\sigma \right]$—the electrical conductivity matrix, *E*—the intensity of the electric field, and $\left[\alpha \right]$—the Seebeck coefficient matrix [33].

The equation of continuity for a dielectric medium is expressed by the Formula (10):

(10) $$D=\;\left[\varepsilon \right]\cdot E$$

where $\left[\varepsilon \right]$—the electric permittivity matrix.

In the absence of a magnetic field, the electric field is non-centrifugal $\left(\nabla \times E=0\right)$ and can be obtained from the electric potential (φ), according to the following equation:

(11) E=−∇φ

Substituting Equations (7) and (8) into Equations (9)–(11) gives a system of coupled thermoelectric Equations (12) and (13):

(12) $$\rho C\frac{\partial T}{\partial t}+\nabla \cdot \left(\left[\Pi \right]\cdot J\right)-\nabla \cdot \left(\left[J\right]\cdot \nabla T\right)=\dot{q}$$

(13) $$\nabla \cdot \left(\left[\varepsilon \right]\cdot \nabla \frac{\partial \varphi }{\partial t}\right)+\nabla \cdot \left(\left[\sigma \right]\cdot \left[\alpha \right]\cdot \nabla T\right)+\nabla \cdot \left(\left[\sigma \right]\cdot \nabla \varphi \right)=0$$

The thermoelectric module (TEG) generates power in response to the temperature difference generated throughout the module [34]. The output power of the TEG module not only depends on the temperature of the heat source and coolant, but also on the resistance of the electrical load.

The electrical power $P_{ele}$ produced by the TEG module is given by Formula (14) [35]:

(14) $$P_{ele}={\left(\alpha_{S}\Delta T_{TEG}\right)}^{2}\frac{R_{L}}{\left(R_{L}+R_{TEG}\right)}$$

where $R_{L}$—the load resistance, $R_{TEG}$—the TEG electrical resistance TEG, $\alpha_{S}$—the Seebeck coefficient, and $\Delta T_{TEG}$—the temperature gradient.

The heat energy *Q_h_* at the hot side of the TEG and the heat energy *Q_c_* at the cold side of the TEG are given by Equations (15) and (16) [36]:

(15) $$Q_{h}=SI_{t}T_{H}+\left(\frac{k_{n}A_{n}}{l_{n}}+\frac{k_{p}A_{p}}{l_{p}}\right)(T_{H}-T_{C})-\frac{1}{2}n\left(\frac{\rho_{p}l_{p}}{A_{p}}+\frac{\rho_{n}l_{n}}{A_{n}}\right)I_{te}^{2}$$

(16) $$Q_{C}=SI_{t}T_{C}+\left(\frac{k_{n}A_{n}}{l_{n}}+\frac{k_{p}A_{p}}{l_{p}}\right)(T_{H}-T_{C})-\frac{1}{2}n\left(\frac{\rho_{p}l_{p}}{A_{p}}+\frac{\rho_{n}l_{n}}{A_{n}}\right)I_{te}^{2}$$

### 2.3. Heat Sink Cooling by Air

The energy balance equation for an air continuous flow system in the heat sink is given by the following equation:

(17) $$\rho_{f}V_{f}C_{P,f}\frac{\partial T_{f}}{\partial t}=h_{}\;\;A_{}\left(T_{TEG}-T_{air}\right)\;-\;\dot{m}C_{p,f}\frac{\partial T_{air}}{\partial x}$$

where x—the direction of air flow, *f*—the fluid in this case air flowing through heat sink, and $\dot{m}$—the inlet mass of air [37].

As the total heat transfer coefficient is a function of the total heat drawn from the system, a Nusselt number calculation can be made there from Equation (18):

(18) $$Nu=\frac{h\cdot D_{h}}{k_{air}}$$

where $k_{air}$—the heat transfer coefficient, $D_{h}$—the effective diameter of the channel, *h*—the heat transfer coefficient in air duct [38].

The relationship between Prandtl and Nusselt numbers is given by Colburn correlation (19):

(19) Nu=0.023Re0.8Pr1/3

The channel friction factor can be computed along the test section by pressure loss Δ*P*, as follow:

(20) $$f=\frac{\Delta P}{(L/D_{h})\cdot \rho \cdot U_{m}^{2}/2}$$

where ρ—the density of air, $U_{m}^{}$—the average velocity of the fluid, *L*—the channel length, and $D_{h}$—the equivalent diameter of the channel.

Buoyancy and free convection can be measured by the ratio of the Grashof and Reynolds number [39]. 

Wind temperature was calculated by following formula:

(21) $$T_{wc}=13.12+0.6215T_{a}-11.37V_{}^{0.16}+0.3965T_{a}V_{}^{0.16}$$

where *T_a_*—the air temperature, *V*—the wind speed, and *T_wc_*—the wind temperature.

The wind temperature was dependent on the wind speed and was equal to 0.1–12 °C.

### 2.4. Geometry and Meshing PV–TEG Hybrid System

**Figure 1:**
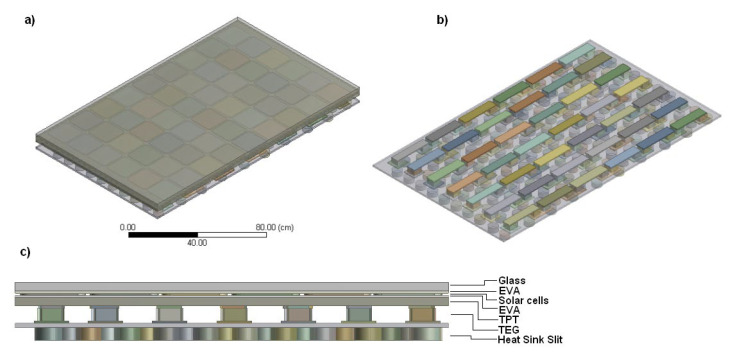
PV–TEG hybrid system model. View of the entire system: (**a**) TEG cells with a cylindrical heat sink, (**b**) front view through the PV–TEG system with of all layers description (**c**).

The 3-dimensional (3-D) model of polycrystalline silicon PV cells covers four layers: glass, ethyl vinyl acetate (EVAx2), silicon solar cells, and TPT (tedlar, PET, tedlar). On the backside of the joined TEG module with the cylindrical fulfillment heat sink, there is a slit to heat removal. The 3-D geometric details of a hybrid PV–TEG module is shown in Figure 1. The solar cell has a dimensions of 156 mm × 156 mm [40,41,42,43]. The thermophysical properties of all the layers are given in Table 1. The optical parameters of a PV module are presented in Table 2.

The model was developed based on a SunLink solar panel with 250-W power PV cells. A PV module has dimensions of 1640 mm × 992 mm × 40 mm. The thermoelectric device module (TEM) is placed on the PV backside to generate electric power by Seebeck or Peltier effects and consists of 42 TEG of p-n doped junctions made of semiconductors, and connected to each other by an electric conductive material (copper). The Seebeck coefficient, as well as electrical and thermal conductivity of Bismuth Telluride (Bi_2_Te_3_) material, depend on the operating temperature. Table 3 lists the basic calculation parameters of one TEG module. Air, which is the cooling source of the TEG system, flows through the cylindrical slit. The temperature-dependent material properties for p-n thermocouples are given in Table 4. The heat sink attached at the back of a TEG is made of high thermal conductivity metal, such as aluminum. At higher heat transfer coefficients, the TEG module is an additional thermal resistance for PV cooling. In low convection coefficients, the TEG helps to improve the device performance, due to the thermal resistance decreasing.

The wind-cooled TEG passive radiator consists of 190 cylinders with a diameter of 0.5 cm and a length of 1–3 cm. Their arrangement was aimed at ensuring the lowest temperature of TEG cell-cold ends.

A numerical mesh was generated with the Ansys Meshing program using the option that creates the highest quality domain division. In this analysis, hexagonal mesh was used. The minimum cell size is 0.001 m for the heat sink and 0.003 m for a PV–TEG module. A grid study was carried out to investigate the independency of the results on the mesh size. This 3-D precise mesh was constructed of 19,582,362 cells; 40,795,293 faces; and 9,502,846 nodes.

A grid convergence approach was carried out to verify the independence of the models. Two additional coarser grids (medium and coarse grid) of the bare gable roof were created. The element numbers of very fine, fine, medium, and coarse grids were around 19 million, 16 million, 12 million, and 8 million, respectively. The average and maximum intensity grids showed similar results; accordingly, the solutions from the maximum intensity grid were considered to be grid independent.

The 3-D numerical model for different thermoelectric element geometries investigated was built for the hybrid PV–TEG system and it was accurately meshed into small hexagonal cells to increase the accuracy and convergence results. The dimensionless wall distance y+ was correspondingly 8, which is finer mesh to long computational.

Good mesh quality was also controlled as skewness > 0.68. The mesh near the layers contact surface was refined. In order to estimate the grid convergence uncertainty of the CFD solution, the study the grid convergence index (GCI) method based on a Richardson extrapolation was employed. For transient analysis with LES, a necessary condition for the convergence of a numerical method for partial differential equations was the numerical/mathematical domain of dependence. This condition is known as the Courant–Friedrichs–Lewy (CFL) number, as mentioned in the formula. The Courant number or CFL was calculated based on velocity, cell-size, and the time step at each cell, and was 0.58. The solutions also needed to be independent of the mesh resolution.

### 2.5. Numerical Approach

A specialized “Solar Ray Tracing” tool from Ansys Fluent software (Ansys Inc., Canonsburg, PA, USA) was employed to simulate a solar radiation. The optical parameters of the photovoltaic panel were determined as the global solar radiation absorption coefficient α = 0.7. Simulations were carried out for an outside temperature of 10–30 °C with a solar radiation intensity ΦS of 200–1200 W/m^2^ (variable step 200 W/m^2^). The convective heat transfer coefficient was determined at α_con_ = 8 W/m^2^·K. Equations of continuity, momentum, and energy were solved using the finite volume method with the second order upwind using the FLUENT computational tool. The numerical simulation was realized using k-ε the Re-Normalization Group (RNG) model of turbulence, recommended for air flow inside channels. The velocity of the air behind the heat sink cooled by air was set at: 1.42 m/s; 3.26 m/s; 3.76 m/s; and 8.47 m/s, suitable for wind conditions in Poland. The SIMPLE method employs a relationship between velocity and pressure corrections to enforce mass conservation and to obtain the pressure field. A fully implicit numerical scheme was employed, in which upwind differences are used for the convective terms and central differences for the diffusion terms. The calculation was iterative, with convergence criteria of 10^−6^ for the energy equation and 10^−3^ for the pressure, velocities, and continuity equations. At the end of each solver iteration, the residual sums for each of the conserved variables were computed. During the numerical procedure, the imbalances (errors) of the discretized equations were monitored and these defects were commonly referred to as the residuals of the system and terminated the numerical process when a specified tolerance was reached. In an iterative numerical solution, the residual will never be exactly zero. However, the lower residual value is the more numerically accurate to the solution. For satisfactory convergence, the residuals should diminish as the numerical process progresses.

### 2.6. Co-Simulation Procedure of a PV/TEG Hybrid Model with a Single-Phase Inverter

**Figure 2:**
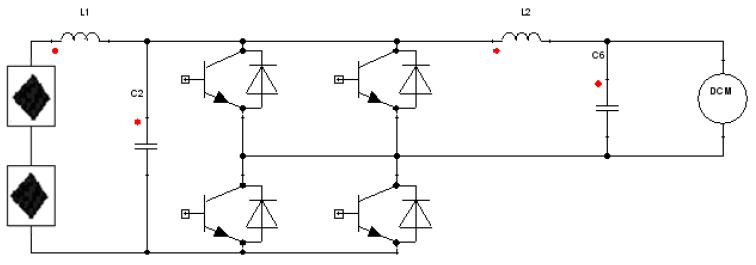
Block diagram of the single-phase H-bridge inverter in co-simulation approach realized by Fluent/Electro-Thermal/Simplorer software.

The Simplorer multiphysics platform software was applied to build a multi-level system model between an inverter and a PV–TEG module. A PV–TEG module has its “via boundary conditions” integrated in its electronic interface. This approach to “coupled software module” of Fluent and Simplorer is called co-simulation and increases the computational time for Fluent transient simulation. The interaction between a multi-physical numerical device models in a non-linear circuitry, which could be studied in a time-efficient way in this approach. A hybrid time synchronization was employed between Simplorer and Fluent software (strategy of optimal digital and conservative analog synchronization). The co-simulation environment (Fluent–Simplorer) enables interaction between a multi-physical numerical CFD models and non-linear components, which could be studied in a time-efficient way. Samanski’s coefficient in Simplorer environment updates and reassesses the Jacobian matrix after a certain number of iterations. An adaptive Trapezoid–Euler algorithm was applied to analyze numerical methods of coupling two multiphysics, i.e., a TEG and PV full transient model, to a single-phase inverter (Figure 2).

## 3. Results and Discussion

An illustration of the applicability of a numerical study of PV–TEG/heat sink cooled by air flow of a proposed co-simulation with single-phase inverter is given based on the results presented in terms of conversion efficiencies of a PV–TEG module as a function of irradiance, wind speed, and ambient temperature, as well as Seebeck and Joule’s heat effects of TEG’s to observe the influence of each parameter on a hybrid model. The Thomson effect was also considered to enable the understanding of TEG temperature variations effects on the material properties.

### 3.1. Results of Heat Sink TEG Cold Leg

**Figure 3:**
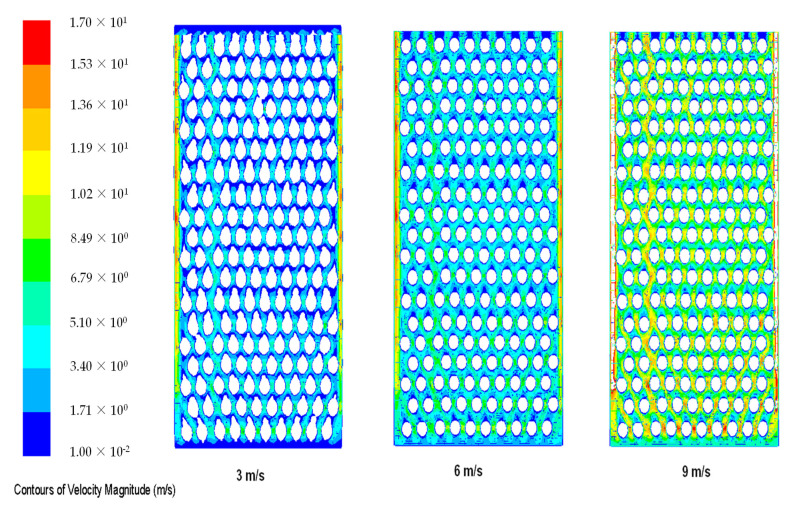
Contours of air velocity in the TEG heat sink.

Figure 3 shows air velocity contours in a TEG heatsink for air velocity inlet = 3; 6; 9 m/s. The convective air coefficient was 7 W/m^2^·K. As the air velocity increased, the rate of heat removed from the cold ends of the TEG increased. In order to achieve the necessary cooling in a cell temperature, heat transfer surface area was increased, forcing air circulation to increase heat transfer convection with turbulence effects. The amount of energy produced by the system is directly related to direct radiation from the sun. The total power generated by the PV–TEG system compared to the stand alone photovoltaic system and generated power increased by 14.3% at STC conditions [2] for a wind speed of 6 m/s in a k-ε turbulence model.

As the wind speed increases in the ducts, the heat transfer rate in heat sink increases. This indicates the dynamic nature of heat transfer from heat sink to air, depending on changing wind speed.

**Figure 4:**
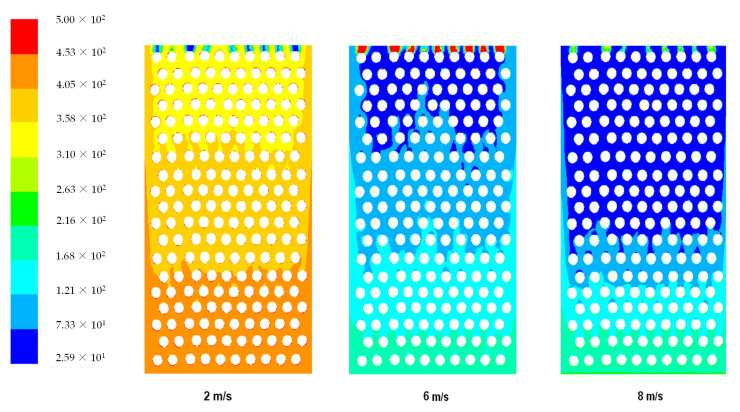
Temperature distribution of TEG heat sink for various wind velocities.

Figure 4 shows temperature distribution for six different air speeds at the heat sink inlet. As illustrated, as the air speed increases, the caused heat transfer coefficient also increases above 4 m/s and cools the heat sink evenly. Moreover, it may be concluded that the air circulation is more effective due to the turbulent air flows. The images obtained from the simulations show velocity contours for three different air velocities. The temperature changes dynamically and dead volume zones can be seen, in which the occurrence of no or very low flow circulation are significantly reduced. The control volume is more intensive and the temperature distribution is more homogeneous. The heat sink reduces the temperature of TEG and maintains the electrical efficiency at higher level. The heat transfer increases with increasing air velocity, and the effectiveness of the friction coefficient decreases. As seen from Figure 3, low air velocity, e.g., 1 m/s, causes high temperature gradients and low heat transfer coefficient. In the case of high wind speeds (6–8 m/s), low temperature gradients occur and the temperature is evened along the entire length of the heat sink. As the wind speed increases, the total temperature gradients disappear, but intensity of a dead volume, where the air gets stuck and is heated up, is significant. The best temperature distribution occurs for high wind speeds.

### 3.2. Solar Cells and TEG Module Results Analysis

**Figure 5:**
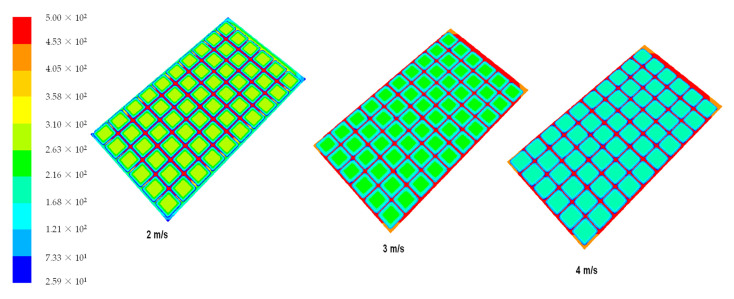
Temperature distribution of PV cells depending on the different wind velocities of TEG heat sink cooling.

Figure 5 presents the temperature of photovoltaic panels depending on the wind speed affecting the TEG heat sink. As the wind speed increases, the amount of heat received from TEG is higher, which significantly increases the efficiency of the PV panel. Wind speed affects the natural convection at the collector surface, therefore, at low wind speeds, less energy is lost due to natural convection and more heat is available for extraction from the back surface of the PV layer, which can no longer be noticed. The calculations took into account the value of incidental solar radiation of 100–1200 W/m^2^, which includes the value of emissions in both summer and winter season. Numerical calculations based on input parameters, i.e., wind speed and solar radiation power, affect the performance parameters of the PV–TEG system. Above the value of 600 W/m^2^ of solar radiation, the efficiency of the PV panel decreases. Numerical calculations show that a wind speed of 6 m/s is enough to cool the PV panel to the temperature of 73 °C (at 1200 W/m^2^ radiation intensity) and that the wind of a speed of 8 m/s cools the PV panel to the temperature of 25 °C, causing an increase in energy on both PV and TEG sides. In the absence of cooling, the temperature of the PV panel at maximum solar radiation may cause self-ignition of the installation as a result of hotspots. For example, at wind speed of 6 m/s, the overall electrical efficiency (PV + TEG) increases by 17.6%, and wind speed of 8 m/s to 23.9%, giving a total energy gain at panel efficiency (22.3%) up to 41.2% in the best conditions. Therefore, the heat absorbed by PV can be dissipated directly without passing throughout the TEG.

**Figure 6:**
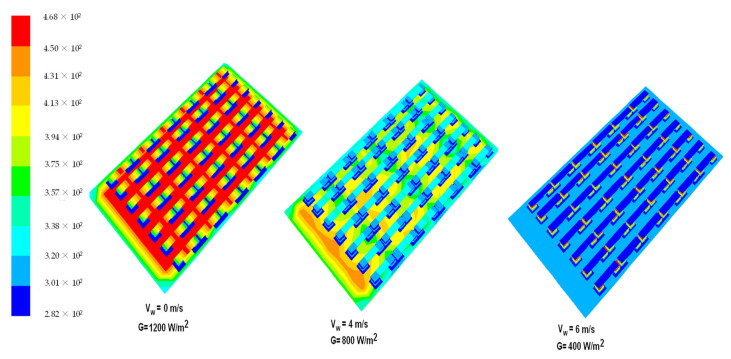
Temperature distribution of TEG heat sink cooling depending on wind velocity.

A total of 42 TEG cells were used in the simulation of the PV panel. The number of cells has a significant impact on the panel’s temperature gradient. The electrical efficiency of the solar panel is increased by the power generated for the same amount of light energy radiation. The temperature change in TEG cells depending on wind speed and solar radiation intensity is shown in Figure 6. As it can be seen, a solar radiation intensity of 1200 W/m^2^ and 0 m/s of wind speed in the heatsink of TEG results in a temperature increase in the system due to the lack of temperature gradient. When a passive radiator is used, the temperature distribution is different. Wind speed and intensity of solar radiation are one of the most important parameters affecting the electrical and thermal efficiency of TEG cells. With the increase in wind speed (2–8 m/s) and solar radiation intensity from 400 to 800 W/m^2^, the efficiency of TEG cells increases along with the temperature gradient. Low heat utilization at a low wind speed (1–2 m/s) contributes to an energy yield of 5–7%, which will significantly increase the payback period.

**Figure 7:**
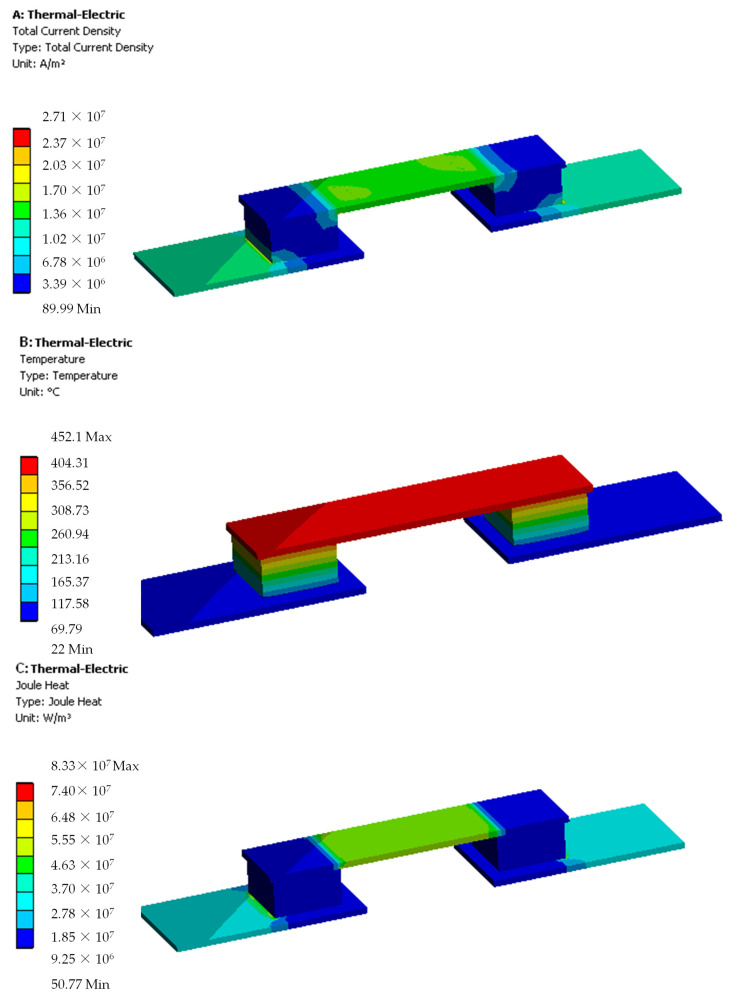
(**A**) Current density, (**B**) temperature distribution, and (**C**) Joule heat on TEG module.

Figure 7 presents a thermoelectric analysis for a single TEG cell for wind speed of 3.83 m/s and solar radiation intensity 600 W/m^2^. Figure 7A shows the distribution of total current density in the layer of a single thermogenerator. Moreover, Figure 7B shows the temperature distribution. The temperature profile exhibits non-linear behavior at higher temperature gradients. This behavior is caused by the internal Joule’s heat generation at the base of the TEG cell. The output power and the conversion efficiency with the Thomson effect included are about 25.3% and 18.1%, respectively, a magnitude lower than those without Thomson effect. It exhibits a relatively good heat transfer enhancement effect on the improvement of TEG net power in PV solar cells. A change in the temperature of the photovoltaic module and the cold side of the TEG module with solar radiation for different wind speeds, taking into account heat losses due to conduction, convection, and radiation in combination with Seebeck effects, causes a change in the value of the PV output power, caused by a different heat conduction coefficient, and affects the overall efficiency of the PV/TEG system.

**Figure 8:**
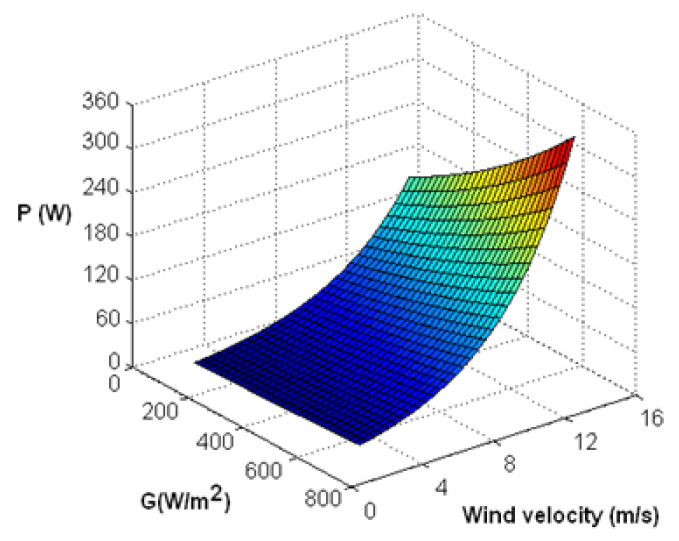
Three-dimensional variation of PV–TEG hybrid system variation of overall power output of the PV–TEG system in a wind velocity function.

Figure 8 presents the overall power output efficiency (PV + TEG) depending on wind speed and solar radiation. As expected, as the wind speed increases, the overall system efficiency increases. In Figure 8, an increase in wind speed and intensity of solar radiation can be seen to cause an increase in power output; however, solar irradiance above 600 W/m^2^ weakens the output power by 6.9% compared to 800-W/m^2^ radiation. The optimum conditions for solar energy production are irradiance of 621 W/m^2^ and wind speed of 11.58 m/s. Figure 8 also presents the relationship between the electrical and thermal efficiencies as a function of wind velocity. It can be seen that, as the wind speed increases, the electrical and thermal efficiencies of the PV panel increase, and the optimal point occurs at the highest possible wind speed, which has a synergistic effect on the PV–TEG system. The heat sink of the TEG module plays a very important role in increasing both electrical and thermal efficiency. However, a higher temperature in PV causes efficiency reduction. The power generation increases, especially at a higher wind speed. The optimum generation point may help design electric circuits that support active cooling in maintaining system stability to ensure an increase in electrical efficiency in the future.

**Figure 9:**
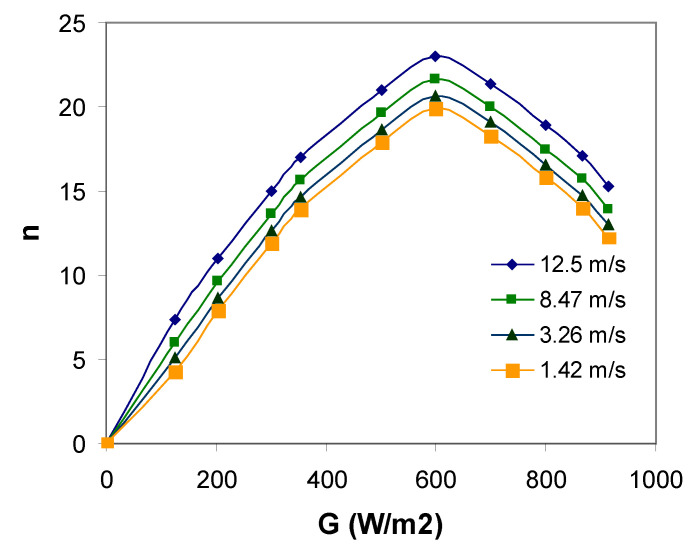
Changes in the value of the efficiency coefficient η of a PV cell as a function of solar radiation intensity G0 (without TEG).

As illustrated in Figure 9, changes in the G0 solar radiation intensity value are accompanied by a change in the conversion efficiency of solar radiation energy to electricity in a photovoltaic cells (PVs). The non-linear nature of changes in the efficiency coefficient η as a function of solar radiation intensity G0 is conditioned by many factors, such as a change in cell temperature and voltage generated by the photovoltaic module based on the numerical model of the photovoltaic cell.

**Figure 10:**
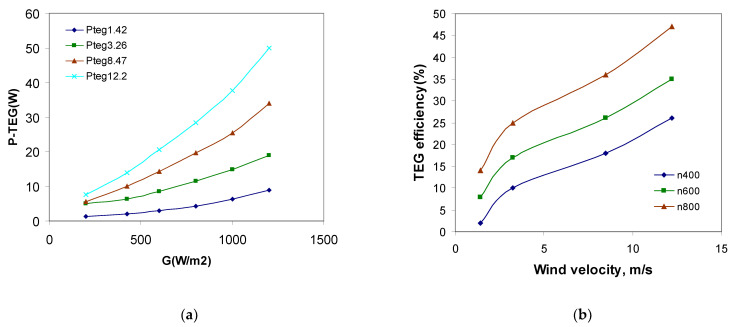
Change in the output power generated by the TEG cell depending on wind speed and solar radiation intensity: (**a**) TEG efficiency depending on wind velocity (1.42; 3.26; 8.47; 12.2 m/s), (**b**) TEG efficiency depending on wind velocity and radiation intensity (400; 600; 800 W/m^2^).

Figure 10a shows TEG power output depending on wind speed and solar radiation intensity. It can be seen that, as the intensity of solar radiation increases, the output power of the TEG module also increases [42]. However, increasing wind speed increases the temperature gradient, which translates into the of the TEG module power output. Therefore, as seen from Figure 10b, as the wind speed increases, the efficiency of the TEG module increases approximately with the second order inertial. The power output of TEG system increases with increase, both in solar radiation and number of thermocouple elements, due to a higher heat input. This is considered to be an important finding that will help in accurately choosing a TEG temperature value and in applying geometrical configuration to reach the maximum efficiency.

**Figure 11:**
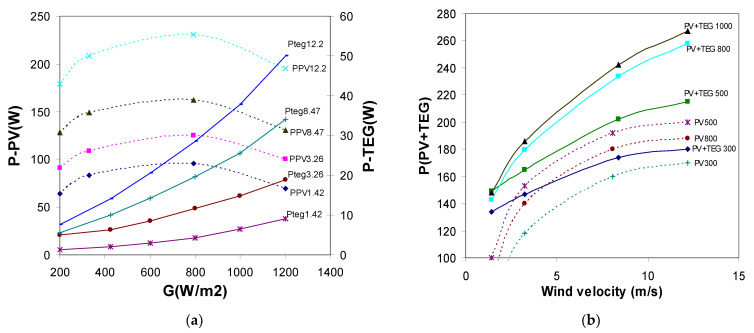
Total power of PV–TEG hybrid system: (**a**) depending on the intensity of solar radiation for selected wind velocity (1.42; 3.26; 8.47; 12.2 m/s), (**b**) depending on wind velocity for selected solar radiation (300, 500, 800, 1000 W/m^2^).

As seen from Figure 11a, the efficiency of TEG system increases as the intensity of solar radiation increases. In the case of the PV solar cell, the optimum solar radiation intensity is 600–800 W/m^2^, and, above that, its output power decreases. Figure 11b shows that the overall efficiency of a hybrid system presents an increasing trend as the thermoelectric element height increases. A synergy effect can be observed in which the heat from the PV panel is absorbed by the TEG cell, increasing its efficiency, which results in better performance for the entire system. Furthermore, the overall efficiency of the PV–TEG system is much higher when compared to a single PV module. Given the Thomson effects, the TEG output power increases with increasing solar radiation and wind speed. Due to the higher efficiency of the TEG cell, the power yield is much higher at a solar radiation power of 1000 W/m^2^, although the efficiency of the PV cell above 600 W/m^2^ decreases.

A good inverter should present technical parameters that will allow its flexible design and operation in a photovoltaic system, with features translating into the efficiency of PV installation. This helps to determine the financial benefits for its users to a greater extent than the phase inverter itself.

## 4. Conclusions

The influence of air passive cooling slit on PV–TEG electrical efficiency and comparison with PV with/without cooling slit, depending on the external conditions such as wind velocity, intensity of solar radiation, or ambient temperature, were analyzed. The multiphysical PV–TEG model (partial differential equation system) can be employed by circuit designers (ordinary differential equations system) to develop and test novel power/control circuit concepts under different operating conditions. In the paper, a novel method for co-simulation of the PV/TEG/inverter, which can be applied in optimization of the energy properties of power electronics systems (inverter) depending on the multiphysics of the PV/TEG system, was presented. The computer simulations performed confirmed the full usefulness of this method in the design. Analysis of high-performance inverters, as well as its applicability in designing this type of system, was extended by additional parasitic elements of the inverter system. Furthermore, PV–TEG models were successfully applied in a co-simulation with electrical power circuitry, which enabled true system-level simulation to test higher frequency transformer inverters, assuming standard network conditions. Furthermore, the co-simulation can be performed to improve inverter co-design, in terms of maintenance and reliability. This approach can be employed by circuit designers to develop and test novel power/control circuit concepts in vector or scalar inverter control.

## Figures and Tables

**Table 1:** Properties of layers employed in the PV module.

Material	Thickness (mm)	Thermal Conductivity (W/m·K)	Specific Heat Capacity (J/kg·K)	Density(kg/m^3^)
Glass	3.2	5 × 10^2^	790	2.45
EVA (x2)	0.5	2.09 × 10^3^	2090	0.95
PV cells	0.4	1.3 × 10^2^	677	2.33
TPT	0.3	1.5 × 10^−1^	1250	1.20

**Table 2:** Optical properties of layers employed in the PV module.

Material	Absorptivity	Reflectivity	Transmissivity	Emissivity
Glass	0.04	0.04	0.92	0.85
EVA (x2)	0.08	0.02	0.9	–
PV cells	0.9	0.08	0.02	–
Tedlar	0.14	0.86	0	0.9

**Table 3:** Calculation parameters of the TEG module.

Parameter	N-Type Semiconductor Leg	P-Type Semiconductor Leg
Thermal conductivity(W/m·K)	1.265	1.011
Resistivity (Ω m)	1.314 × 10^−5^	1.119 × 10^−5^
Seebeck coefficient α (V/K)	−1.721 × 10^−4^	2.037 × 10^−4^
Currency (A)	11.1	10.09

**Table 4:** Temperature dependent material properties.

Material Type	Thermal Conductivity k (W/m·K)	Seebeck Coefficient S (V/K)
p-type	0.0000361558T^2^ − 0.026351342T + 6.22162	(0.003638095T^2^ + 2.74380952T − 296.214286) × 10^−6^
n-type	0.0000334549T^2^ − 0.0244586622T + 5.625893	(0.00153073^2^ − 1.08058874T − 28.3889024) × 10^−6^

## Data Availability

The data presented in this study are available on request from the corresponding author.

## Nomenclature

$c_{p}$	specific heat capacity (kJ/kg·K)
*T*	temperature (K)
$\dot{q}$	volumetric solar energy absorption (W/m^3^)
$P_{gen}$	electric power generation by volume (W/m^3^)
λ	thermal conductivity (W/m·K)
$\eta_{ref}$	reference cells efficiency
$T_{PV}$	temperature of PV cells (K)
$T_{R}$	reference operating temperature (K)
β	coefficient represents amount of efficiency loss per each temperature degree rise in PV cells (K^−1^)
A	exposed area of PV cells (m^2^)
$r_{i}$	reflectivity
$V_{i}$	volume of the layer
$G_{i}$	solar radiation intensity (W/m^2^)
*Q_PV-GC_*	heat transfer between glass cover and photovoltaic cells
*Q_gca_, Q_gcs_*	heat transfer between glass cover and PV cells, ambient air and surrounding environment
C	heat capacity(J/kg·K)
$\dot{q}$ *_h_*	heat generation rate per unit volume(W/m^3^)
∇	Nabla operator
q	heat flux (W/m^2^)
J	electric current density vector (A/m^2^)
*D*	electric flux density vector (C/m^2^)
*E*	electric field intensity (V/m)
$R_{TEG}$	TEG electrical resistance (Ω)
$R_{L}$	load resistance (Ω)
$\Delta T_{TEG}$	temperature gradient (K)
$\dot{m}$	inlet mass of air (kg/s)
$D_{h}$	effective diameter of the channel (m)
*h*	heat transfer coefficient in air duct (W/m^2^·K)
$U_{m}^{}$	average velocity of the fluid (m/s)
**Greek letters**	
*α*	Seebeck coefficient matrix (V/K)
η	efficiency
*λ_qc_*	absorption rate of glass cover (W/m·K)
ρ	density (kg/m^3^)
ε	turbulence dissipation rate (m^2^·s^−3^)
μ	viscosity (Pa·s)
$\mu_{tl}$	liquid phase turbulent viscosity (m^2^·s)
$\mu_{ts}$	solid phase kinematic eddy viscosity (m^2^·s^−1^)
*σ*	surface tension
$\sigma_{t}$	turbulent Prandtl number
$\sigma_{k}$	turbulence model constant for the k-equation
$\sigma_{\varepsilon }$	k-ε turbulence model constants
Π	matrix of Peltier coefficient (V)
$\left[\sigma \right]$	electrical conductivity matrix (S/m)
$\left[\alpha \right]$	Seebeck coefficient matrix (V/K)
$\left[\varepsilon \right]$	electric permittivity matrix (F/m)
